# lncRNA MALAT1 participates in metformin inhibiting the proliferation of breast cancer cell

**DOI:** 10.1111/jcmm.16742

**Published:** 2021-06-24

**Authors:** Yongye Huang, Ziyan Zhou, Jin Zhang, Zhenzhen Hao, Yunhao He, Zihan Wu, Yiquan Song, Kexun Yuan, Shanyu Zheng, Qi Zhao, Tianye Li, Bing Wang

**Affiliations:** ^1^ College of Life and Health Sciences Northeastern University Shenyang China; ^2^ School of Computer Science and Software Engineering University of Science and Technology Liaoning Anshan China

**Keywords:** autophagy, ER stress, MALAT1, metformin

## Abstract

In recent years, the repurposing of conventional and chemotherapeutic drugs is recognized as an alternative strategy for health care. The main purpose of this study is to strengthen the application of non‐oncological drug metformin on breast cancer treatment in the perspective of epigenetics. In the present study, metformin was found to inhibit cell proliferation, promote apoptosis and induce cell cycle arrest in breast cancer cells at a dose‐dependent manner. In addition, metformin treatment elevated acH3K9 abundance and decreased acH3K18 level. The expression of lncRNA MALAT1, HOTAIR, DICER1‐AS1, LINC01121 and TUG1 was up‐regulated by metformin treatment. In metformin‐treated cells, MALAT1 knock‐down increased the Bax/Bcl2 ratio and enhanced p21 but decreased cyclin B1 expression. The expression of Beclin1, VDAC1, LC3‐II, CHOP and Bip was promoted in the cells received combinatorial treatment of metformin and MALAT1 knock‐down. The reduced phosphorylation of c‐Myc was further decreased in the metformin‐treated cells in combination with MALAT1 knock‐down than metformin treatment alone. Taken together, these results provide a promising repurposed strategy for metformin on cancer treatment by modulating epigenetic modifiers.

## 1 INTRODUCTION

Cancer remains as a global public health problem and is the second most common cause of death from illness.^1^ Among them, breast cancer is the most common cancer and second leading form of cancer death in women around the world.^1^, ^2^ Cancer is characterized as a complex disease involving abnormal cell growth and has the ability of invasion and/or metastasis. It should point out that cancer is a kaleidoscopic disease, the occurrence and development of which could be ascribed to genetic, epigenetic, metabolic and environmental factors.^3^, ^4^ Breast cancer is also a high heterogeneous disease including three different subtypes: luminal, HER2‐positive and triple‐negative (TNBC). These characteristics result in enhancing the difficulty and complexity of cancer therapy. Recently, it has been shown that drug repurposing and drug combinations could be an alternative means with few side effects and low costs to improve the outcome of cancer therapy.

Metformin is one of the most widely used oral glucose‐lowering drugs for the treatment of type 2 diabetes. In recent years, metformin exhibits anticancer property in a variety of cancers, including breast cancer.^3^, ^5^ For example, metformin has been shown to inhibit cell proliferation via decreasing the N6‐methyladenosin (m6A) level in breast cancer.^6^ It could also conquer the resistance to gemcitabine by reversing epithelial‐mesenchymal transition (EMT) progress via modulating DNA methylation of miR‐663 in pancreatic cancer.^7^ In addition, metformin treatment induces cell apoptosis and reduces histone H3 lysine 27 trimethylation (H3K27me3) in ovarian cancer.^8^ Thus, it seems that utilizing the epigenetic effect of metformin could broaden its application on cancer therapy.

Epigenetic regulation includes DNA methylation, histone modification, non‐coding RNAs (ncRNAs) and other chromatin structure alterations. Long non‐coding RNA (lncRNA), a subtype of RNA with a length of more than 200 nucleotides that has little or no protein‐coding potential, has attracted growing attention in cancer treatment. They act as key factors in regulating the proliferation, apoptosis and metastasis of cancer cells. LncRNA metastasis‐associated lung adenocarcinoma transcript 1 (MALAT1), also known as nuclear‐enriched abundant transcript 2 (NEAT2), is widely studied in several types of cancer.^9^ MALAT1 is one of the first identified cancer‐related lncRNA, and increasing evidences point out that it acts as a biomarker of numerous cancers, including breast cancer.^10^ A variety of studies have found the aberrant expression of MALAT1 in many cancer types, and the overexpression of which is shown to be associated with metastasis and poor survival.^11^, ^12^ It would be reasonable to hypothesis that MALAT1 is a potential target for cancer therapy.

Epigenetics is closely associated with carcinogenesis, cancer development and establishment of chemoresistance. Epigenetic modifiers have garnered interest as novel targets for developing anticancer drugs. Therefore, we tried to enhance the anti‐tumour outcome of metformin by modulating epigenetic modifier in this study.

## 2 MATERIALS AND METHODS

### 2.1 Cell culture and treatments

The human breast cancer cells MCF7 used in this study was obtained from the Shanghai Zhong Qiao Xin Zhou Biotechnology Co., Ltd. Cells were maintained in Dulbecco's modified Eagle's medium (DMEM) containing 10% foetal bovine serum (FBS), 1% glutamine, 1% nonessential amino acids and 100 U/mL penicillin/streptomycin in a humidified incubator at 37°C under a 5% CO_2_ atmosphere.

Metformin was purchased from Selleck Chemicals and dissolved in ddH_2_O at a concentration of 5 M for storage and diluted to varied concentrations in cell culture medium for cell treatments.

### 2.2 Cell viability assay

The cell viability was assayed over 4 days using a cell counting kit‐8 (CCK‐8) according to the manufacturer’ instructions. MCF7 cells were seeded at a density of 5 × 10^3^ cells per well into 96‐well plates with three wells being used for each assayed group, and cultured for CCK‐8 assay after treating with metformin (0, 5, 10, 20 and 40 mmol/L). Ten microliters of CCK‐8 solution were added to each well at the indicated time‐point, after which the plate was incubated for 2 h at 37°C. The absorbance value in each well was measured at 450 nm by microplate reader.

### 2.3 Apoptosis detection using flow cytometry

Apoptotic cells were measured using fluorescein isothiocyanate (FITC) conjugated Annexin V/propidium iodide (PI) staining. Briefly, 3 × 10^5^ cells were seeded in a 6‐cm dish and incubated for 12 hours at 37°C. Cells were then treated with metformin as mentioned above and incubated in the incubator for 24 hours more. Cells were collected and incubated with Annexin V‐FITC and PI for 15 minutes with binding solution in the dark at room temperature. Live and dead cells were quantified by flow cytometry (BD Biosciences) using a FITC signal detector and a PI signal detector. Data were collected for 10,000 cells per sample and presented as the percentage.

### 2.4 Determination of cell cycle distribution

Cell cycle analysis was performed as previous described.^13^ In brief, cells at a density of 3 × 10^5^ cells were plated in a 6‐cm dish and incubated for 12 hours at 37°C. Cells were then treated with increasing concentrations (0, 5, 10 and 20 mmol/L) of metformin for 24 hours. After treatment, cells were harvested using 0.1% trypsin in 2.5 mmol/L EDTA, rinsed twice with ice‐cold PBS, collected into single‐cell suspension and fixed with 70% ethanol at −20°C overnight. Before flow cytometry analysis, cells were washed with ice‐cold PBS and suspended in PI/RNase Staining Buffer Solution (BD Biosciences) in the dark at room temperature for 15 minutes. The fluorescence‐activated cells were determined by a flow cytometer (Fortessa, BD Biosciences), and data were analysed with ModFit LT software.

### 2.5 RNA isolation and quantitative RT‐PCR analysis (qPCR)

Total RNA was isolated from treated MCF7 cells using TRIzol reagent (Tiangen Biotech) and was reverse‐transcribed into cDNA using the All‐in‐One cDNA synthesis SuperMix (Bimake). The primers’ sequences were listed in Table S1. Quantitative RT‐PCR was conducted using 2× SYBR Green qPCR Master Mix (Bimake) with glyceraldehyde phosphate dehydrogenase (GAPDH) used as an internal control on a CFX96 Real‐time PCR detection system.

### 2.6 Western blotting

Protein extraction was performed in cell lysis buffer supplemented with a protease inhibitor cocktail. The concentration of extracted protein was measured using a bicinchoninic acid (BCA) protein assay kit (Beyotime). Equal amounts of protein (20 μg) from each sample were electrophoresed on 12% sodium dodecyl sulphate polyacrylamide gel electrophoresis (SDS‐PAGE). The separated proteins were transferred to polyvinylidene difluoride (PVDF) membranes (Millipore, MA, USA), which were then blocked in 5% nonfat milk solution at room temperature for 90 minutes. Blot membranes were washed with TBST buffer and incubated with primary antibodies (Table S2) overnight at 4°C. After washing with TBST, the membranes were incubated with appropriate HRP‐conjugated secondary antibody for 1 hour at room temperature. ECL florescence was then developed, and protein bands were captured on an ECL detection system.

### 2.7 Cell migration assay

Transwell assay was applied to evaluate migration ability. After treatment with metformin (0, 5, 10 and 20 mmol/L), 2 × 10^4^ MCF7 cells were suspended in serum‐free medium and seeded into the upper chamber of each well (200 μL per chamber). And then 600 μL of 10% FBS‐containing medium was added to the lower chamber. Post‐incubation at 37°C for 24 hours, the chambers were taken out and washed with PBS. The residual cells on the upper membranes were erased using a cotton swab. The remaining cells were fixed with methanol for 30 minutes at room temperature and stained with 0.2% crystal violet for 20 minutes. After washing with PBS, five fields of view were randomly selected and observed under an inverted optical microscope (Leica) to count the cells.

### 2.8 siRNA transfection

The lncRNA MALAT1 siRNA sequence was designed based on a previous study^14^ and chemically synthesized by GenePharma. When MCF7 cells growing into 70% confluence, oligonucleotides were transfected using Lipofectamine 3000 reagent (Invitrogen) according to the manufacture's guidelines. After being incubated with MALAT1 siRNA for 24 hours, cells were re‐transfected for another 24 hours. Cells were washed and discarded transfection reagents. Subsequently, cells were treated with 20 mmol/L metformin and incubated for another 24 hours. Cells were harvested and subjected into qPCR and Western blotting.

### 2.9 Statistical analysis

The statistical analyses were conducted using SPSS and GraphPad prism software. Statistically significant differences were determined using one‐way analysis of variance (ANOVA). Quantitative data were shown as the mean ± SEM. A level of *P* < .05 was considered statistically significant.

## 3 RESULTS

### 3.1 Metformin inhibited breast cancer cell viability in a dose‐dependent manner

**FIGURE 1:**
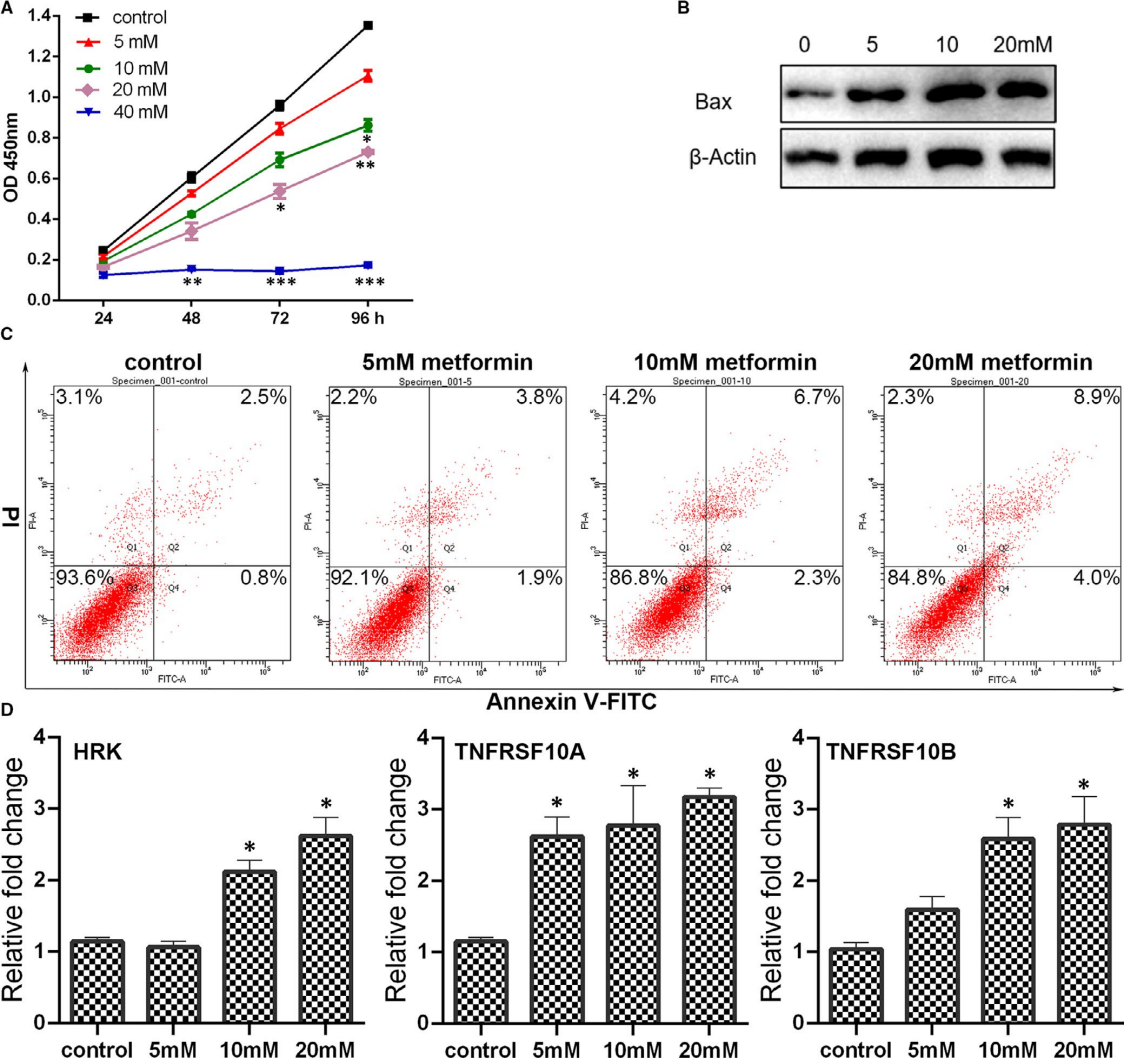
Metformin altered the proliferation and apoptosis of breast cancer cells. (A) The cell proliferation of breast cancer cells determined by CCK‐8 assay. (B) Expression of Bax in cells with metformin treatment by Western blot analysis. Grayscale analysis for Western blot could be seen in Figure S4. (C) Apoptosis in the metformin‐treated cells was assessed by Annexin V/PI staining using flow cytometry. (D) Expression of apoptosis‐associated genes in metformin‐treated cells was analysed by qPCR (mean ± SEM of duplicate experiments). *P < .05 vs control, **P < .01 vs control and ***P < .001 vs control

The effect of metformin on breast cancer cell proliferation was firstly determined. Cell viability examined by CCK‐8 assay was used as a measure of cell proliferation. Breast cancer cells MCF7 were treated with metformin at different concentration (0, 5, 10, 20 and 40 mmol/L) for 24, 48, 72 and 96 hours. As shown in Figure 1A, an increasement of the optical density (OD) values was observed in the cells treated with metformin at the same concentration gradient (0, 5, 10 and 20 mmol/L) from 24 to 96 hours. However, there is no significant difference of the OD values in the cells treated with 40 mmol/L metformin among different time‐points, possibly indicating that high concentration of metformin inhibited cell proliferation seriously. Furthermore, at the same time‐point, the OD values in the cells treated with high concentration of metformin were much lower than those treated with low concentration of metformin. In sum, cell densities were decreased obviously by metformin treatment at a dose‐dependent manner.

### 3.2 Metformin‐induced apoptosis of breast cancer cells

The flow cytometry using Annexin V‐FITC/PI staining was applied to evaluate the apoptosis rate of MCF7 cells which were treated with different concentration of metformin. As presented in Figure 1C, metformin promoted MCF7 cells apoptosis rate at a dose‐dependent manner. In order to further confirm the occurrence of apoptosis induced by metformin treatment, the expression of apoptosis‐related genes was calculated. As the metformin concentration increasing, the expression of HRK, TNFRSF10A and TNFRSF10B was enhanced as revealed by qPCR. In addition, the Bax protein expression level increased significantly in the cells treated with metformin (5, 10 or 20 mmol/L), especially in the 10 mmol/L group and 20 mmol/L group. Generally, high concentration of metformin could trigger apoptosis of MCF7 cells remarkedly.

### 3.3 Metformin‐triggered cell cycle arrest of breast cancer cells

**FIGURE 2:**
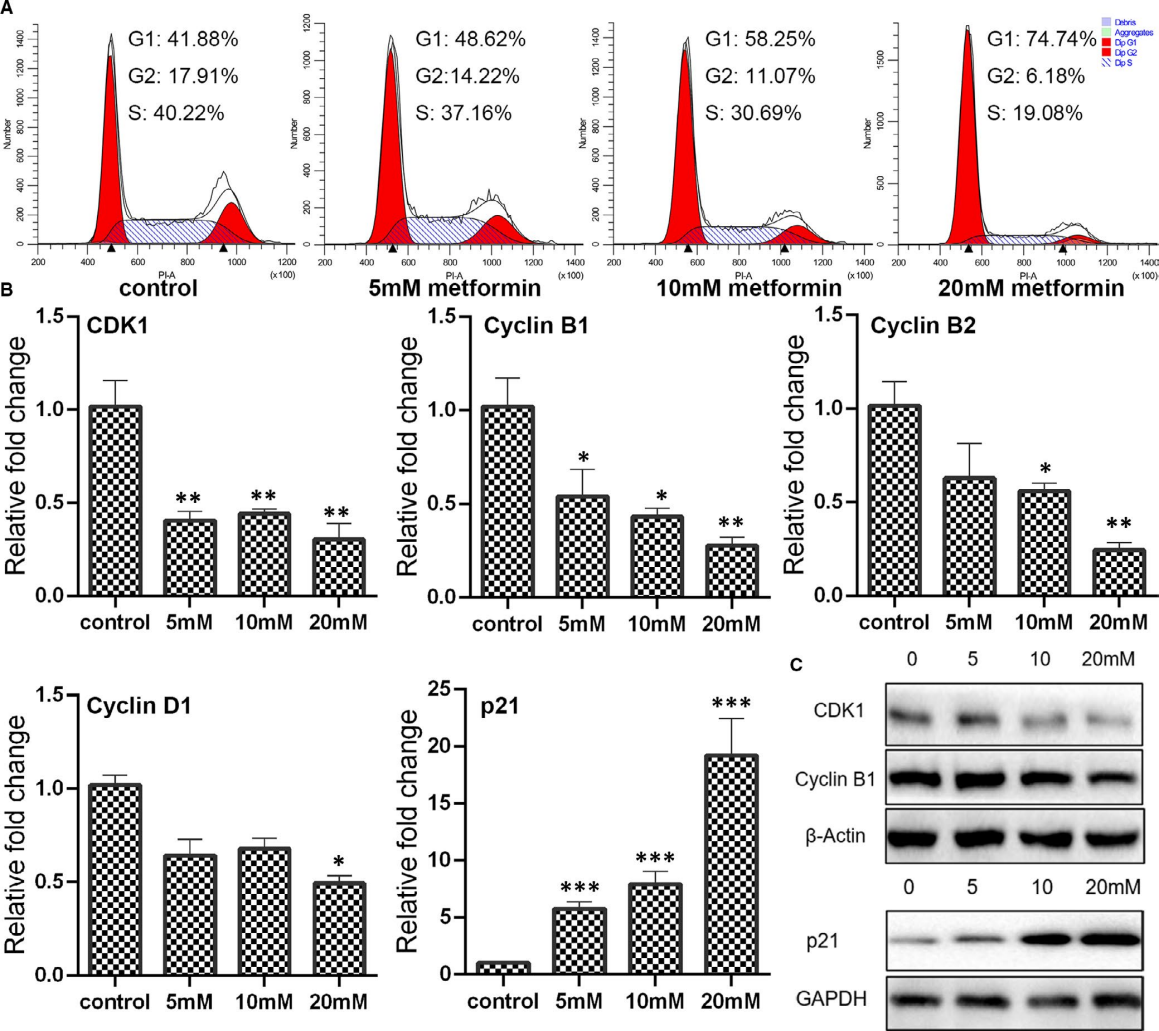
Determination of cell cycle progress in metformin‐treated cells. (A) Cell cycle distribution in breast cancer cells treated with metformin for 24 h was measured by propidium iodide (PI) staining using flow cytometry. (B) Expression of cyclins and cyclin‐dependent kinases as determined by qPCR (mean ± SEM of duplicate experiments). *P < .05 vs control, **P < .01 vs control and ***P < .001 vs control. (C) Expression of CDK1, cyclin B1 and p21 as evaluated by Western blot analysis. Grayscale analysis for Western blot could be seen in Figure S4

Apoptosis is always compatible with cell cycle arrest. To exploit the effect of metformin treatment on cell cycle distribution, flow cytometry was conducted. The results revealed that after being treated with metformin at 5, 10 or 20 mmol/L, the cell accumulation percentage in G0/G1 phase was at 48.62%, 58.25% and 74.74%, compared with 41.88% in the control group. The cell decreased percentage in G2/M phase was at 14.22%, 11.07% and 6.18%, compared with 17.91% in the control group. Furthermore, the cell decreased percentage in S phase was at 37.16%, 30.69% and 19.08%, compared with 40.22% in the control group. The results conferred that metformin could trigger G0/G1 phase cell cycle arrest in MCF7 cells. The CDKN1A (p21), acts as the regulator of G1/S and G2 check points, has been found as a tumour suppressor in many types of cancer. Both qPCR and Western blotting analysis showed that metformin up‐regulated the expression of p21, especially in the 20 mmol/L metformin group (Figure 2B,C). The p21 is also a negative regulator of G1‐CDK1. As expected, the expression of CDK1 was significantly down‐regulated by metformin exposure (Figure 2B,C). In addition, metformin also decreased the expression of cyclin B1, cyclin B2 and cyclin D1 at a dose‐dependent manner (Figure 2B,C).

### 3.4 Cellular homeostasis was altered by metformin treatment

**FIGURE 3:**
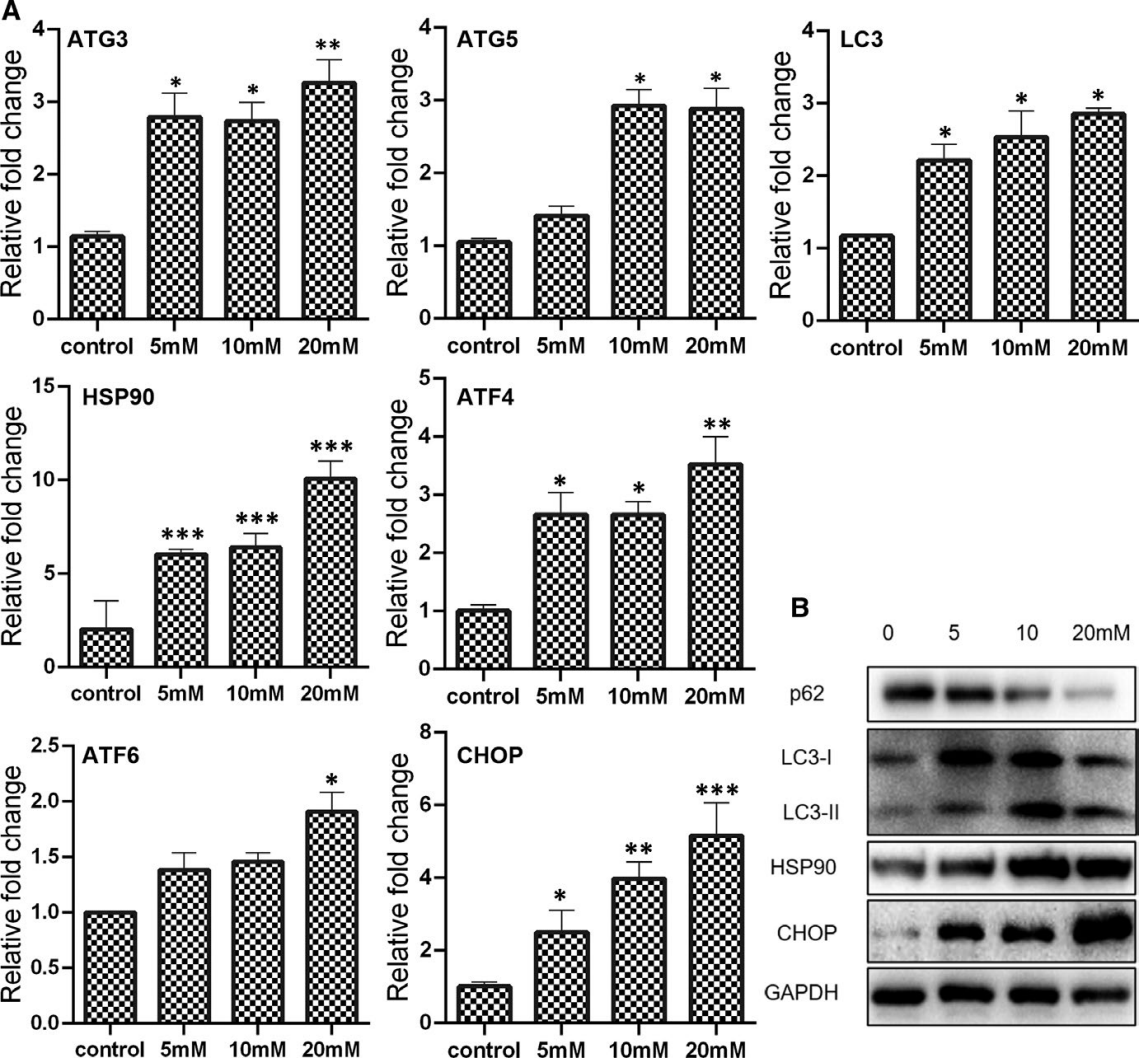
Expression of autophagy and ER stress‐related genes in breast cancer cells treated with metformin for 24 h. (A) mRNA expression as determined by qPCR (mean ± SEM of duplicate experiments). *P < .05 vs control, **P < .01 vs control and ***P < .001 vs control. (B) Protein expression as measured by Western blot analysis. Grayscale analysis for Western blot could be seen in Figure S4

Autophagy is a highly conserved and fundamental cellular process that important for maintaining cellular homeostasis. As shown in Figure 3A, metformin induced the up‐regulation in expression of ATG3, ATG5 and LC3 mRNA. Results of Western blotting analysis also found that LC3‐II expression was promoted in the cells treated with metformin (Figure 3B). Furthermore, the expression of p62/SQSTM‐1, an autophagy receptor known to participate in proteasomal or autophagosomal protein degradation, was gradually decreased in the cells treated with metformin from 0 to 20 mmol/L (Figure 3B), further confirming that metformin triggered autophagy at a dose‐dependent manner. Heat‐shock proteins (HSP) are conserved chaperones crucial for protein degradation, maturation and refolding, to maintain protein homeostasis. It was found that higher expression of HSP90 presented in the cells treated with 5, 10 or 20 mmol/L metformin, compared with the control group (Figure 3). Altering ER homeostasis leads to accumulation of misfolded or unfolded proteins and induces an event named ER stress. To investigate whether ER stress was involved in metformin treated, the expression of ATF4, ATF6 and CHOP was determined. Results showed that the expression of all ER stress‐related genes examined in this study was increased after metformin exposure (Figure 3). Especially, the expression of CHOP was largely promoted in the cells treated with 20 mmol/L metformin. These results possibly indicated that the cellular homeostasis of breast cancer cells was disturbed by metformin treatment.

### 3.5 Metformin‐suppressed cell migration by blocking Wnt/β‐catenin signalling

**FIGURE 4:**
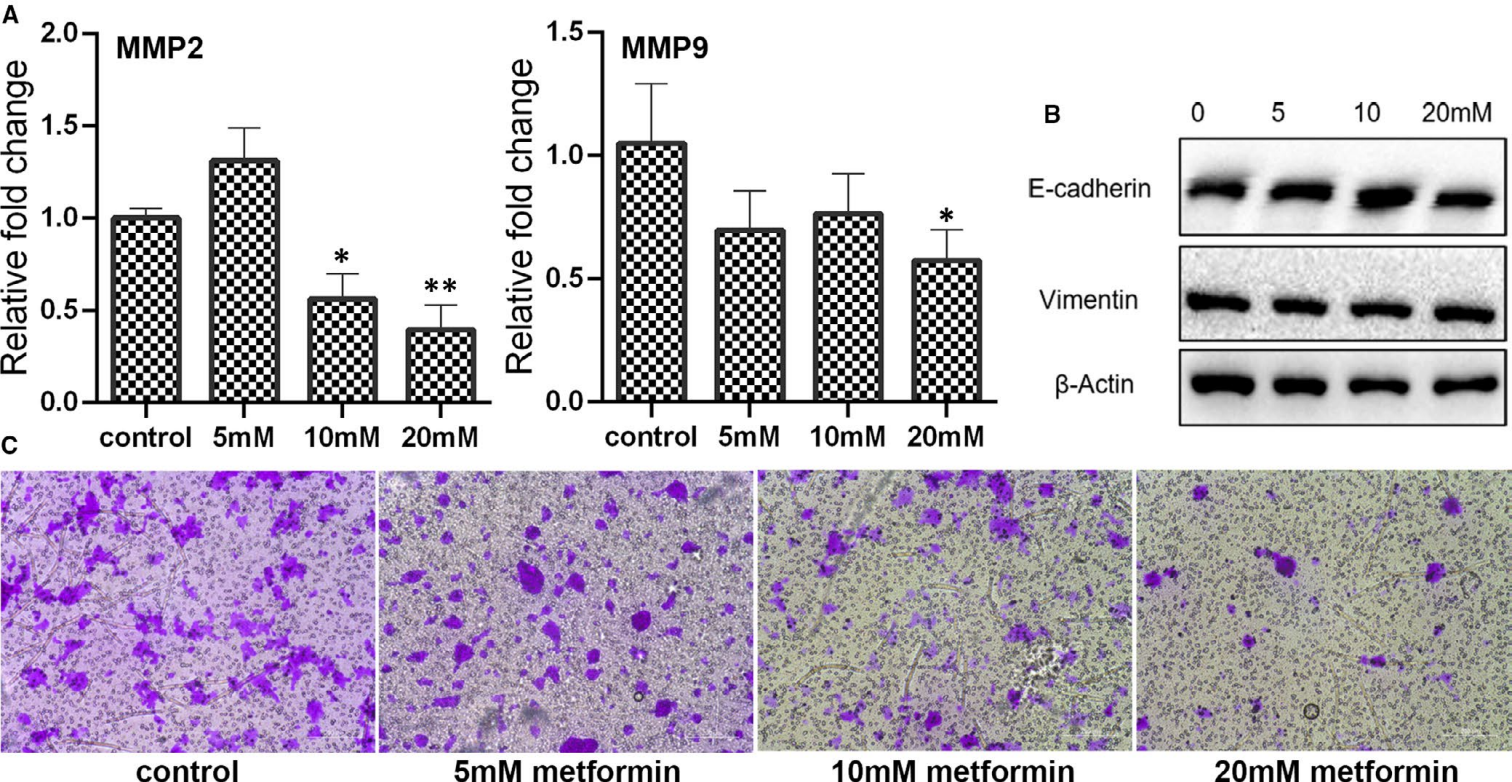
Examination of breast cancer cell migration after treating with metformin for 24 h. (A) Expression of MMP2 and MMP9 as examined by qPCR (mean ± SEM of duplicate experiments). *P < .05 vs control, **P < .01 vs control and ***P < .001 vs control. (B) Expression of E‐cadherin and Vimentin as accessed by Western blot analysis. Grayscale analysis for Western blot could be seen in Figure S4. (C) Cell migration as measured by Transwell assay

Matrix metalloproteinases (MMPs) exert critical function in tumour cell invasiveness and metastasis. As shown in Figure 4A, the expression of MMP2 was decreased in the cells treated with 10 or 20 mmol/L metformin, and the expression of MMP9 was significant down‐regulated in the 20 mmol/L metformin group. However, there is no significant difference on the expression of E‐cadherin and Vimentin between control and metformin‐treated group (Figure 4B). To further unveil the impact of metformin treatment on cancer cells migration, transwell assay was performed. Results of transwell assay revealed that the migration was inhibited by metformin in a dose‐dependent manner (Figure 4C).

The Wnt/β‐catenin is also found to be associated with tumour cell proliferation, apoptosis, cell cycle regulation, migration and metastasis.^15^ As shown in Figure S1A, the expression of Wnt3a, Wnt5a and β‐catenin was significant down‐regulated in the cells treated with 20 mmol/L metformin. Results of Western blotting further confirmed that the Wnt5a was decreased by 20 mmol/L metformin (Figure S1B). In addition, the expression of β‐catenin was lower in the metformin‐treated cells (5, 10 and 20mM) than the control cells. The expression of c‐Myc, a target gene of Wnt/β‐catenin signalling, was also down‐regulated in the cells treated with 20 mmol/L metformin (Figure S1A). It is possible that metformin inhibited cell migration was associated with decreased Wnt/β‐catenin signalling.

### 3.6 Epigenetic fluctuation in metformin‐treated cells

Epigenetics play an important role in cancer development, progression and therapy. Previous studies have found that metformin could alter the DNA methylation, RNA methylation and histone methylation in cancer cells. Thus, the impact on some epigenetic regulators by metformin treatment was examined in this study. As shown in Figure S2, the expression of RNA binding protein RBBP4, involved in histone acetylation and chromatin assembly, was significantly increased after metformin treatment. The mRNA expression of G9a, a histone methyltransferase, was down‐regulated in the cells treated with 20 mmol/L metformin. Western blotting analysis also confirmed that the expression of G9a was decreased in the metformin‐treated cells (Figure S1B). However, there was no significant difference on the expression of RBBP7, SET and SETD1A among the examined groups. Furthermore, the abundance of histone acetylation was evaluated by Western blotting. An enhanced acetylation of Lys‐9 residues of histone H3 (acH3K9) but decreased acetylation of Lys‐18 residues of histone H3 (acH3K18) were observed in the cells treated with metformin (Figure S2B). These results indicated that the metformin could alter the distribution of histone modifiers in breast cancer cells.

**FIGURE 5:**
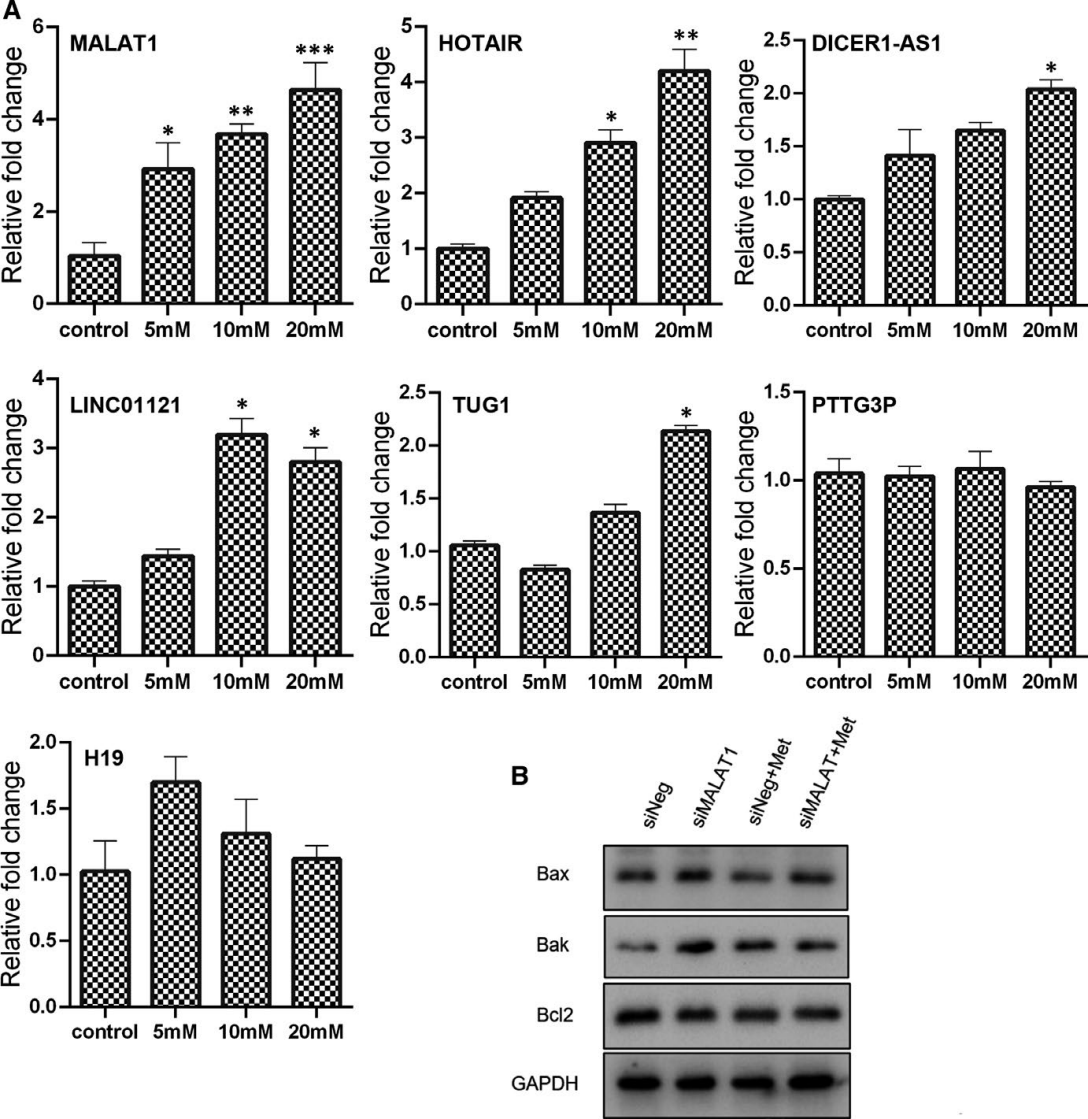
Role of lncRNA in metformin‐treated cells. (A) Expression of lncRNA in metformin‐treated cells as measured by qPCR (mean ± SEM of duplicate experiments). *P < .05 vs control, **P < .01 vs control and ***P < .001 vs control. (B) Expression of apoptosis‐associated genes in cells treated with metformin (20 mmol/L) in combination with MALAT1 knock‐down as determined by Western blot analysis. Grayscale analysis for Western blot could be seen in Figure S5. siNeg, cells treated with negative control siRNA; siMALAT1, cells treated with MALAT1 siRNA; siNeg+Met, cells treated with negative control siRNA in combination with metformin; siMALAT+Met, cells treated with MALAT1 siRNA in combination with metformin

As an important component of epigenetics, lncRNA has long been implicated in the cell growth of several cancers and their aggressive phenotypes. To uncover the role of lncRNA in metformin treatment, we examined the expression of lncRNA MALAT1, HOTAIR, DICER1‐AS1, LINC01121, TUG1, PTTG3P and H19. As shown in Figure 5A, metformin significantly promoted the expression of MALAT1, even at the concentration of 5 mmol/L. As for the expression of HOTAIR and LINC01121, they were enhanced in the cells treated with 10 mmol/L or 20 mmol/L metformin. The expression of DICER1‐AS1 and TUG1 was just increased by 20 mmol/L metformin treatment. Furthermore, there was no significant difference on the expression of PTTG3P and H19 between the metformin treatment and control group. These results indicated that many lncRNA might exert critical function in metformin treatment.

### 3.7 lncRNA MALAT1 knock‐down enhanced the anti‐tumour effect of metformin

**FIGURE 6:**
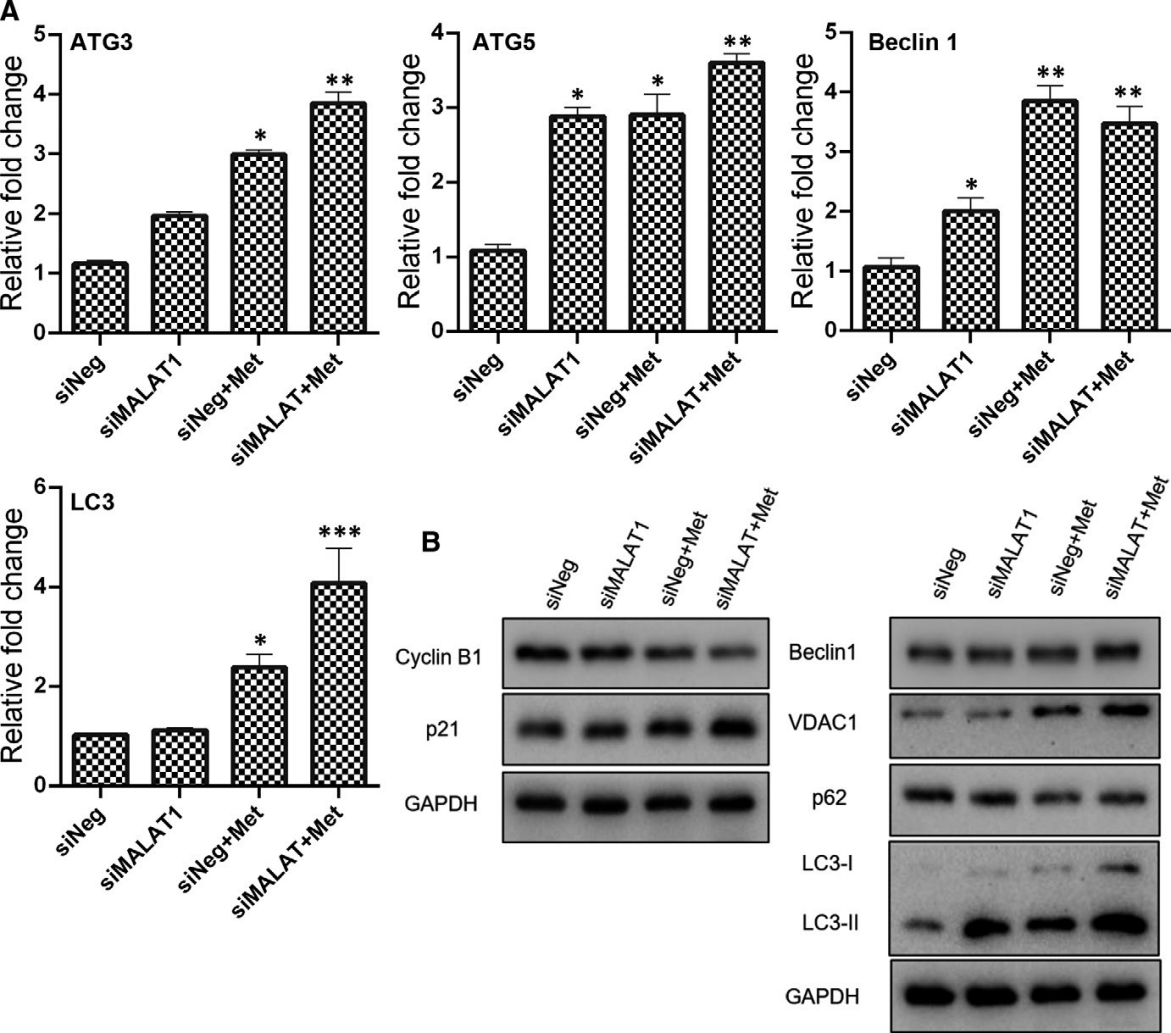
Expression of cell cycle distribution and autophagy induction related genes in metformin (20 mmol/L) treated cells in combination with MALAT1 knock‐down. (A) mRNA expression as determined by qPCR (mean ± SEM of duplicate experiments). *P < .05 vs control, **P < .01 vs control and ***P < .001 vs control. (B) Protein expression as determined by Western blot analysis. Grayscale analysis for Western blot could be seen in Figure S6. siNeg, cells treated with negative control siRNA; siMALAT1, cells treated with MALAT1 siRNA; siNeg+Met, cells treated with negative control siRNA in combination with metformin; siMALAT+Met, cells treated with MALAT1 siRNA in combination with metformin

The lncRNA MALAT1 is involved in cancer development and drug resistance. Therefore, we tried to further uncover the role of MALAT1 in cancer cell viability under metformin treatment. As revealed by Western blotting, the expression of apoptosis‐related genes Bax and Bak were higher in the metformin‐treated cell with MALAT1 knock‐down than metformin treatment alone (Figure 5B). In addition, the ratio of Bax/Bcl2 in the cells with combination treatment of metformin and MALAT1 siRNA was 1.58‐fold higher than that in the control cells, indicating that metformin‐induced apoptosis could be enhanced after MALAT1 knock‐down. As for the expression of cell cycle regulators, cyclin B1 expression was further down‐regulated in the metformin‐treated cells in combination with MALAT1 knock‐down than treatment alone (Figure 6B). In contrast, the incensement of p21 in the metformin‐treated cells was further increased when the MALAT1 had been knock‐down.

The expression of ATG3, ATG5 and LC3 in the cells received combinatorial treatment of metformin and MALAT1 knock‐down was highest among the examined groups (Figure 6A). As revealed by qPCR, metformin treatment in combination with MALAT1 knock‐down significantly promoted the expression of Beclin 1 mRNA. The results of Western blotting revealed that the expression of Beclin 1 and VDAC1 proteins in the cells with combinatorial treatment of metformin and MALAT1 knock‐down were highest among the examined groups (Figure 6B). In addition, the metformin‐treated cells in combination with MALAT1 knock‐down displayed a higher degradation of SQSTM1/p62 protein than the control cells. As expected, metformin in combination with MALAT1 knock‐down further enhanced the expression of LC3‐II in the MCF7 cells, indicating that metformin‐induced autophagy could be strengthened by knocking down the expression of MALAT1. To determine the impact on ER homeostasis after interfering the expression of MALAT1, the expression of Bip, CHOP and XBP1 was evaluated (Figure S3). Results of both qPCR and Western blotting showed that MALAT1 knock‐down further strengthened the metformin‐induced increasement of CHOP and Bip. Most importantly, the spliced form of XBP1 in the cells received combinatorial treatment of metformin and MALAT1 knock‐down was highest among the tested groups, indicating that the metformin triggered ER stress was further exacerbated in the context of MALAT1 knock‐down.

**FIGURE 7:**
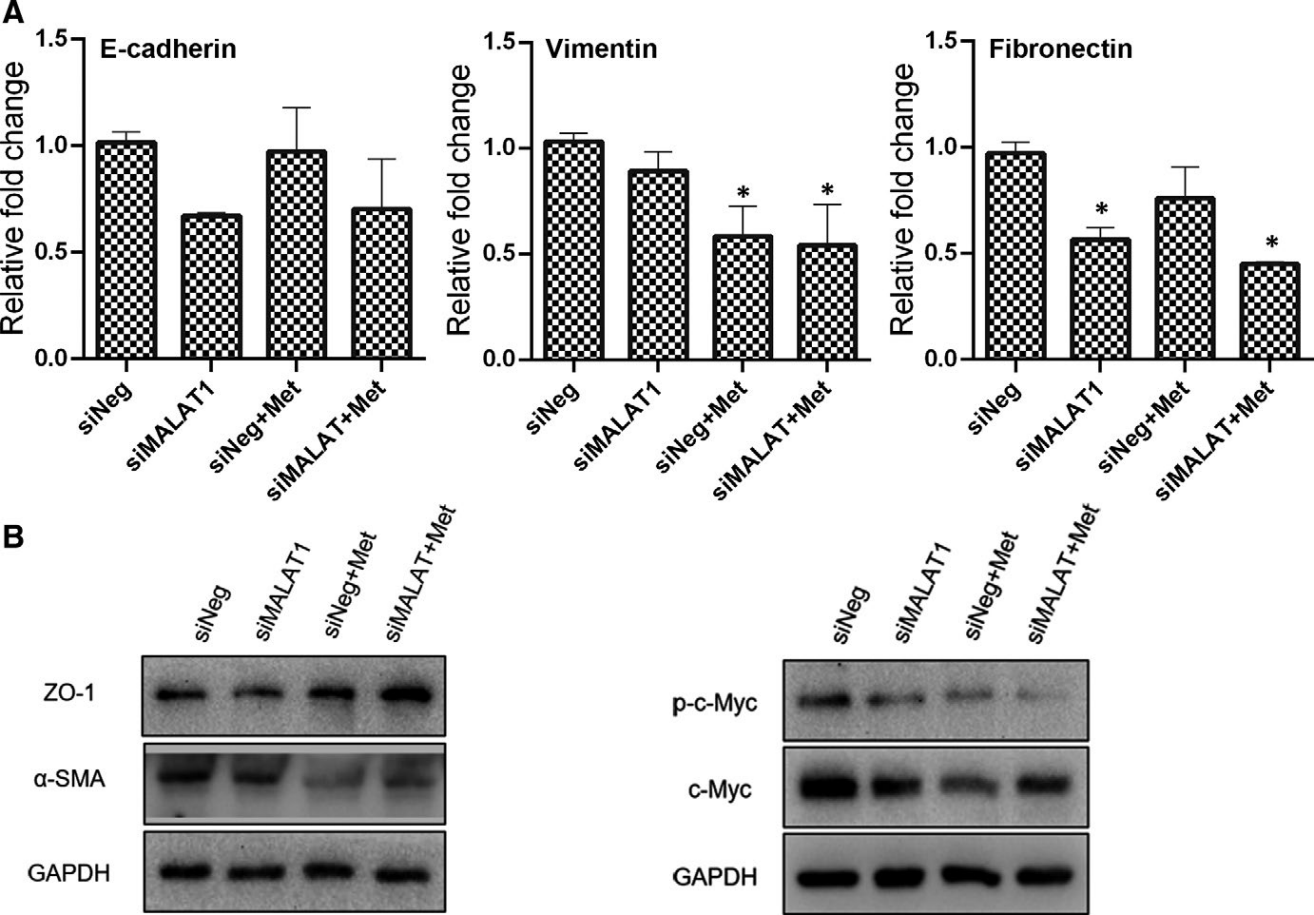
Expression of EMT associated genes in metformin (20 mmol/L) treated cells in combination with MALAT1 knock‐down. (A) mRNA expression as accessed by qPCR (mean ± SEM of duplicate experiments). *P < .05 vs control. (B) Protein expression as measured by Western blot analysis. Grayscale analysis for Western blot could be seen in Figure S5. siNeg, cells treated with negative control siRNA; siMALAT1, cells treated with MALAT1 siRNA; siNeg+Met, cells treated with negative control siRNA in combination with metformin; siMALAT+Met, cells treated with MALAT1 siRNA in combination with metformin

Considering the function of MALAT1 in metastasis, the expression of EMT associated genes were then determined (Figure 7). There was no significant difference on the expression of E‐cadherin among the control, MALAT1 knock‐down, metformin treatment alone and combinatorial treatment. The metformin‐treated cells with MALAT1 knock‐down manifested significant lower expression of Vimentin and Fibronectin than the control cells. Results of Western blotting analysis exhibited that high expression of ZO‐1 and low expression of α‐SMA were presented in the cells received metformin treatment and MALAT1 knock‐down. Furthermore, the reduced phosphorylation of c‐Myc at Serine 62 (S62) phosphorylation site in metformin‐treated cells was further decreased after MALAT1 knock‐down. These findings possibly convey a message that knock‐down of MALAT1 could enforce the ability of migration inhibition of metformin.

## 4 DISCUSSION

The present study found that metformin could inhibit cell proliferation, induce apoptosis and trigger cell cycle arrest in breast cancer cells. Importantly, metformin was showed to alter the expression of several lncRNA, particularly lncRNA MALAT1. The expression of MALAT1 was down‐regulated even by 5 mmol/L metformin. MALAT1, an adverse prognostic factor, up‐regulated expression of which is associated with cancer development and lymph node metastasis in breast cancer.^16^ It seems that there is still possibility of proceeding metastasis for the breast cancer cells escaped from metformin treatment, though fewer migrated cells were observed after metformin treatment via transwell assay. In addition, high expression of MALAT1 is always related to chemosensitivity. In cisplatin‐resistant ovarian cancer tissues and cells, MALAT1 was reported to be increased and knock‐down of which could decrease cisplatin resistance.^17^ In renal cell carcinoma, sunitinib resistance is showed to be positive associated with dramatical up‐regulation of MALAT1, and the chemoresistance could be reversed by MALAT1 knock‐down.^18^ Therefore, regulating the expression of MALAT1 would be a valuable means to repurposing metformin in breast cancer treatment.

Apoptosis, a process of programmed cell death, is divided into extrinsic apoptosis and intrinsic apoptosis in response to either extracellular or intracellular signal.^19^ The extrinsic pathway is usually mediated by cell death receptor (Fas), ligand (Fas L) and tumour necrosis factor (TNF) superfamily receptors. The intrinsic apoptosis is primarily regulated by Bcl2 family genes, including pro‐apoptotic Bcl‐2 members (ie Bax and Bak) and anti‐apoptotic Bcl2 members (ie Bcl2). In the present study, apoptosis was induced by metformin at a dose‐dependent manner. Meanwhile, the expression of Bax, Bak, HRK, TNFRSF10A and TNFRSF10B was increased in the metformin‐treated cells, indicating that metformin could possibly activate both extrinsic apoptosis and intrinsic apoptosis. Several reports have confirmed that MALAT1 knock‐down could trigger activation of apoptosis in cancer cells.^20^, ^21^ In the present study, it also found that the expression of pro‐apoptotic Bcl‐2 members was further enhanced in the metformin‐treated cells with MALAT1 knock‐down, indicating that metformin treatment in combination with MALAT1 knock‐down is more conducive to apoptosis induction than metformin treatment alone.

Besides apoptosis induction, cell cycle regulation is another crucial event for the inhibition of cancer cell survival. Cell cycle, consists of G1, S, G2 and M phase, is accurately controlled by cyclins and cyclin‐dependent kinases (CDKs).^22^, ^23^ In response to mitogenic stimulation, cyclin‐CDK complexes regulate the entrance of cell cycle from resting phase, development through the G1 phase and progression from G1 into S phase.^23^, ^24^ Cyclin B1 is the major partner of CDK1, and cyclin B2 can also combine with CDK1.^25^ In the metformin‐treated cells, increased G0/G1 phase and decreased S phase and G2/M phase were presented at a dose‐dependent manner. The expression of CDK1, cyclin B1, cyclin B2 and cyclin D1 was also down‐regulated by metformin treatment, further confirming that metformin could modulate cell cycle progress. In addition, the expression of p21, encoded by CDKN1A gene, was significantly elevated by metformin treatment. Actually, p21 was initially considered as a cyclin‐dependent kinase regulator to suppress G1/S cell cycle phase.^26^ Recently, increasing evidences have revealed the important role of p21 in cancer development via p53‐dependent and p53‐independent pathways.^26^ In this study, the increased expression p21 of metformin‐treated cells was further promoted after MALAT1 knock‐down. Considering the critical role of p21 in regulating the G1/S and G2 check points, it is not surprising that p21 is evoked by metformin treatment.

Autophagy is an evolutionarily conserved process for maintaining cellular homeostasis through the degradation or recycling of damaged long‐lived proteins, protein aggregates and organelles. Upon autophagy induction, a family of autophagy‐related (ATG) proteins are recruited to form a series of membrane structures, including autophagosomes.^27^ Results of present study showed that the expression of ATG3, ATG5 and LC3‐II was increased in the metformin‐treated cells, indicating that autophagy was involved with the anti‐tumour effect of metformin. However, numerous studies have demonstrated that autophagy executes a dual duty in cancer.^27^, ^28^ Autophagy was initially thought to play a tumour‐suppression role to limit the neoplastic process by eliminating abnormal proteins and/or organelles. Under some conditions, autophagy could also at as a tumour promoter by reducing metabolic stress and enabling cancer cell survival. It should be noted that excessive autophagy would lead to autophagic cell death.^29^ The LC3‐II was further accumulated in the cells received both metformin treatment and MALAT1 knock‐down. Possibly, autophagic cell death also occurred under metformin treatment. Besides autophagy, ER homeostasis is also important for maintaining cell fitness. In general situation, autophagy can mitigate ER stress to maintain ER homeostasis.^30^ However, if the ER stress is continuous or aggravated, cancer cells would be failed to re‐establish ER homeostasis thus inducing apoptosis.^31^ The elevated ER stress by metformin treatment was shown to be further up‐regulated when knocking down the expression of MALAT1. These results possibly indicated that MALAT1 knock‐down could subject more cells into death than metformin treatment alone.

Wnt/β‐catenin signalling is also directly associated with cellular homeostasis, and there is frequent crosstalk between autophagy and Wnt/β‐catenin signalling.^32^ Abnormal regulation of the Wnt/β‐catenin pathway could be in favour of tumour initiation, invasion, metastasis, recurrence and relapse.^33^ Specially, current literature also indicates that inhibiting epithelial‐mesenchymal transition (EMT) process is always connected to inactivation of the Wnt/β‐catenin.^34^, ^35^, ^36^ In the present study, the expression of some components of Wnt/β‐catenin signalling was down‐regulated by metformin treatment. Meanwhile, metformin showed to reduce cancer cell migration. Possibly, these inhibition on cell migration by metformin was also related to Wnt/β‐catenin inactivation in breast cancer cells. Furthermore, it is well known that the protein stability of c‐Myc oncoprotein could be regulated by its conserved threonine 58 (T58) and S62 phosphorylation sites.^37^ In this study, metformin alone or in combination with MALAT1 knock‐down was shown to decrease the c‐Myc protein by dephosphorylating it at S62. In fact, MALAT1 was found to sponge miR‐22 thus counteracting its inhibitory effect on c‐myc and c‐myc‐mediated EMT process in ovarian cancer.^38^ These findings demonstrated that metformin in combination with MALAT1 regulation through modulating Wnt/β‐catenin signalling could serve as a novel strategy for cancer treatment.

Besides the role of MALAT1 in metformin treatment, some other epigenetic regulations would also critical for the anti‐tumour effect of metformin. In fact, metformin regulating DNA methylation, RNA methylation and histone methylation has already documented.^6^, ^7^, ^8^ This study further discovered that the abundance of RBBP4, G9a, acH3K9 and acH3K18 were changed by metformin treatment, suggesting that there might be a crucial role of histone modifiers for metformin cancer therapy. Actually, up‐regulation of HOTAIR, which binds lysine‐specific demethylase 1 (LSD1) and polycomb repressive complex 2 (PRC2),^39^ was observed in the metformin‐treated cells. In addition, metformin was also showed to modulate the HOTAIR promoter methylation pattern in triple‐negative aggressive breast cancer cell line MDA‐MB‐231.^40^ Therefore, modulating epigenetic regulators would be helpful to the repurposing metformin on breast cancer therapy.

## CONFLICT OF INTEREST

The authors declare that they have no competing interests.

## AUTHOR CONTRIBUTIONS

**Yongye Huang:** Conceptualization (equal); Formal analysis (equal); Writing‐original draft (equal); Writing‐review & editing (equal). **Ziyan Zhou:** Investigation (equal). **Jin Zhang:** Investigation (equal). **Zhenzhen Hao:** Investigation (equal). **Yunhao He:** Investigation (equal). **Zihan Wu:** Investigation (equal). **Yiquan Song:** Investigation (equal). **Kexun Yuan:** Investigation (equal). **Shanyu Zheng:** Investigation (equal). **Qi Zhao:** Data curation (equal); Writing‐review & editing (equal). **Tianye Li:** Formal analysis (equal); Investigation (equal); Writing‐review & editing (equal). **Bing Wang:** Conceptualization (equal); Writing‐review & editing (equal).

## Supporting information

Fig S1‐S6

Tab S1

Tab S2

## Data Availability

Data used to support the findings of this study are available from the corresponding author upon request.
