# Supplementary material for: lncRNA MALAT1 participates in metformin inhibiting the proliferation of breast cancer cell
Source: J Cell Mol Med. 2021 Jun 24;25(15):7135–45. doi: 10.1111/jcmm.16742 (PMC8335702; doi:10.1111/jcmm.16742)

lncRNA MALAT1 participates in metformin inhibiting the proliferation of breast cancer cell

Running Title: MALAT1 in metformin treatment

Yongye Huang [1], Ziyan Zhou [1], Jin Zhang [1], Zhenzhen Hao [1], Yunhao He [1], Zihan Wu[1], Yiquan Song [1], Kexun Yuan [1], Shanyu Zheng [1], Qi Zhao [2,*], Tianye Li [1,*], Bing Wang [1,*]

[1] College of Life and Health Sciences, Northeastern University, Shenyang, 110169, China;
[2] School of Computer Science and Software Engineering, University of Science and Technology Liaoning, Anshan, 114051, China.

[*] To whom correspondence should be addressed: Tel: +86-24-83656116. Fax: +86-24-83656116. E-mail: zhaoqi@lnu.edu.cn (Qi Zhao), kafeidou1991@163.com (Tianye Li), wangbing@mail.neu.edu.cn (Bing Wang).

Figure S1. Expression of Wnt signaling genes in metformin treated cells. (A) mRNA expression as determined by qPCR (mean ± SEM of duplicate experiments). (B) Protein expression as examined by Western blot analysis. (C) Densitometry of (B) was calculated by comparing with internal protein β-actin. *$p<0.05$ versus control, and **$p<0.01$ versus control.

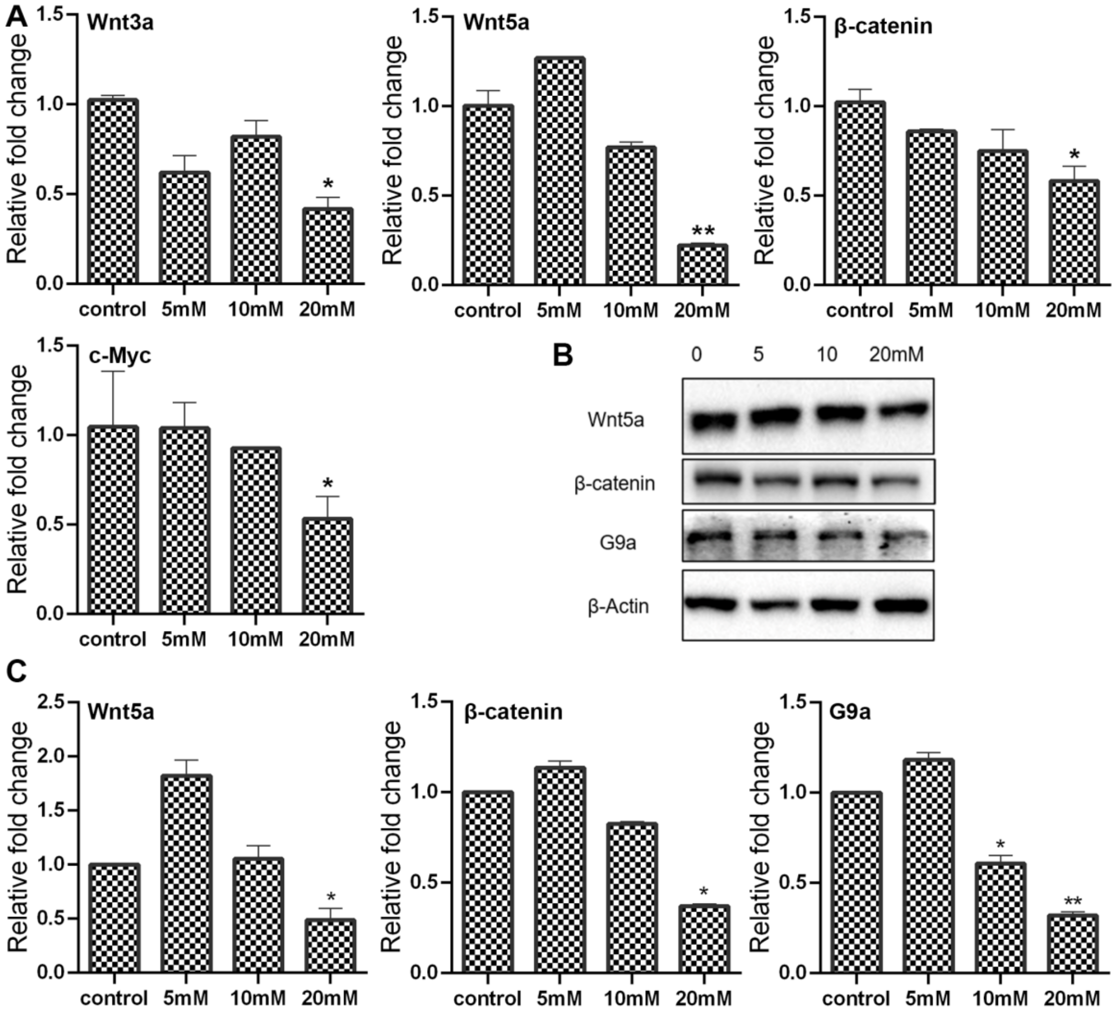

Figure S2. Expression of epigenetic genes in metformin treated cells. (A) mRNA expression as determined by qPCR (mean ± SEM of duplicate experiments). (B) Protein expression as examined by Western blot analysis. (C) Densitometry of (B) was calculated by comparing with internal protein H3. *p<0.05 versus control, **p<0.01 versus control and ***p<0.001 versus control.

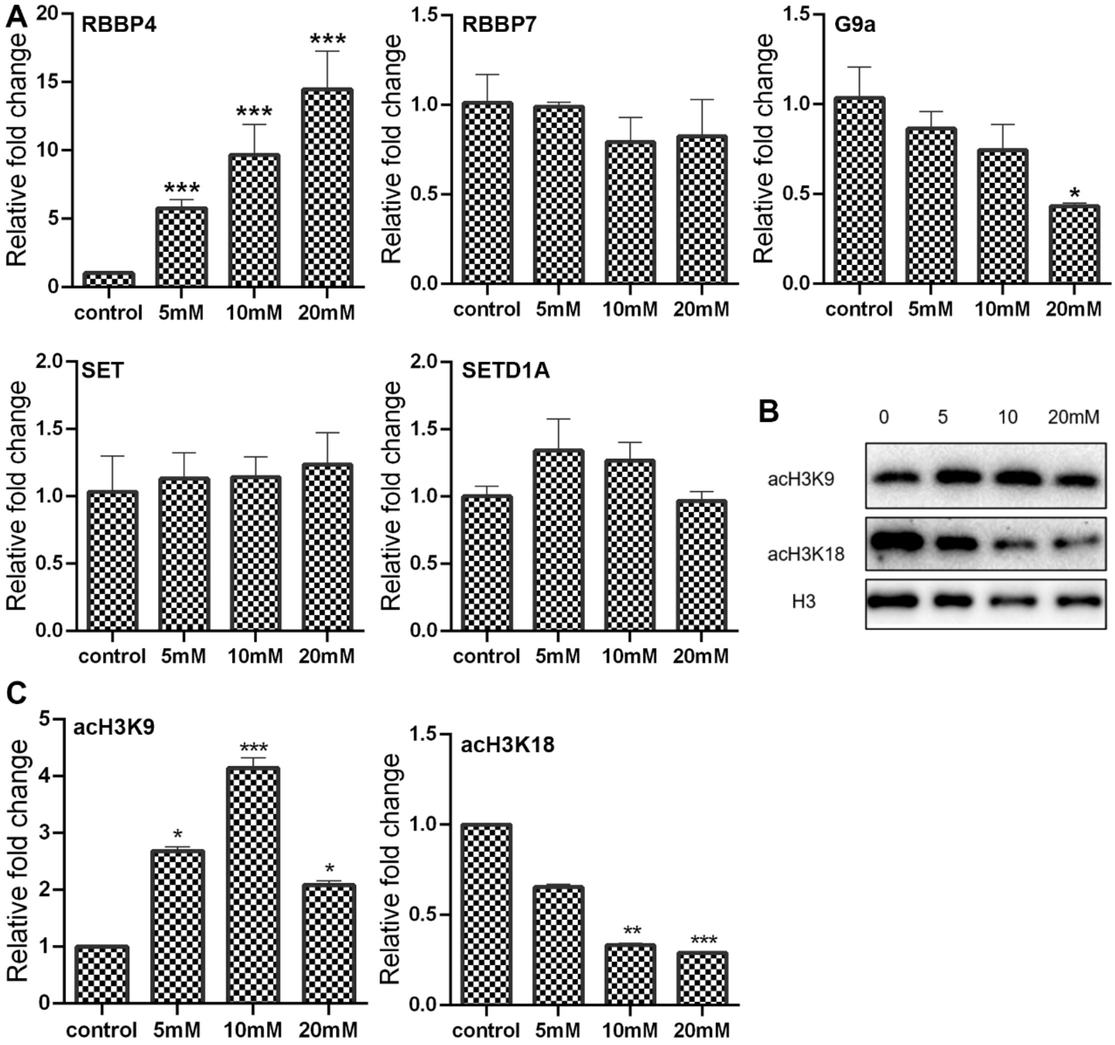

Figure S3. Expression of ER stress related genes in metformin treated cells in combination with MALAT1 knockdown. (A) mRNA expression as accessed by qPCR (mean ± SEM of duplicate experiments). (B) Protein expression as measured by Western blot analysis. (C) Densitometry of (B) was calculated by comparing with internal protein GAPDH. *p<0.05 versus control, **p<0.01 versus control and ***p<0.001 versus control. siNeg, cells treated with negative control siRNA; siMALAT1, cells treated with MALAT1 siRNA; siNeg+Met, cells treated with negative control siRNA in combination with metformin; siMALAT+Met, cells treated with MALAT1 siRNA in combination with metformin.

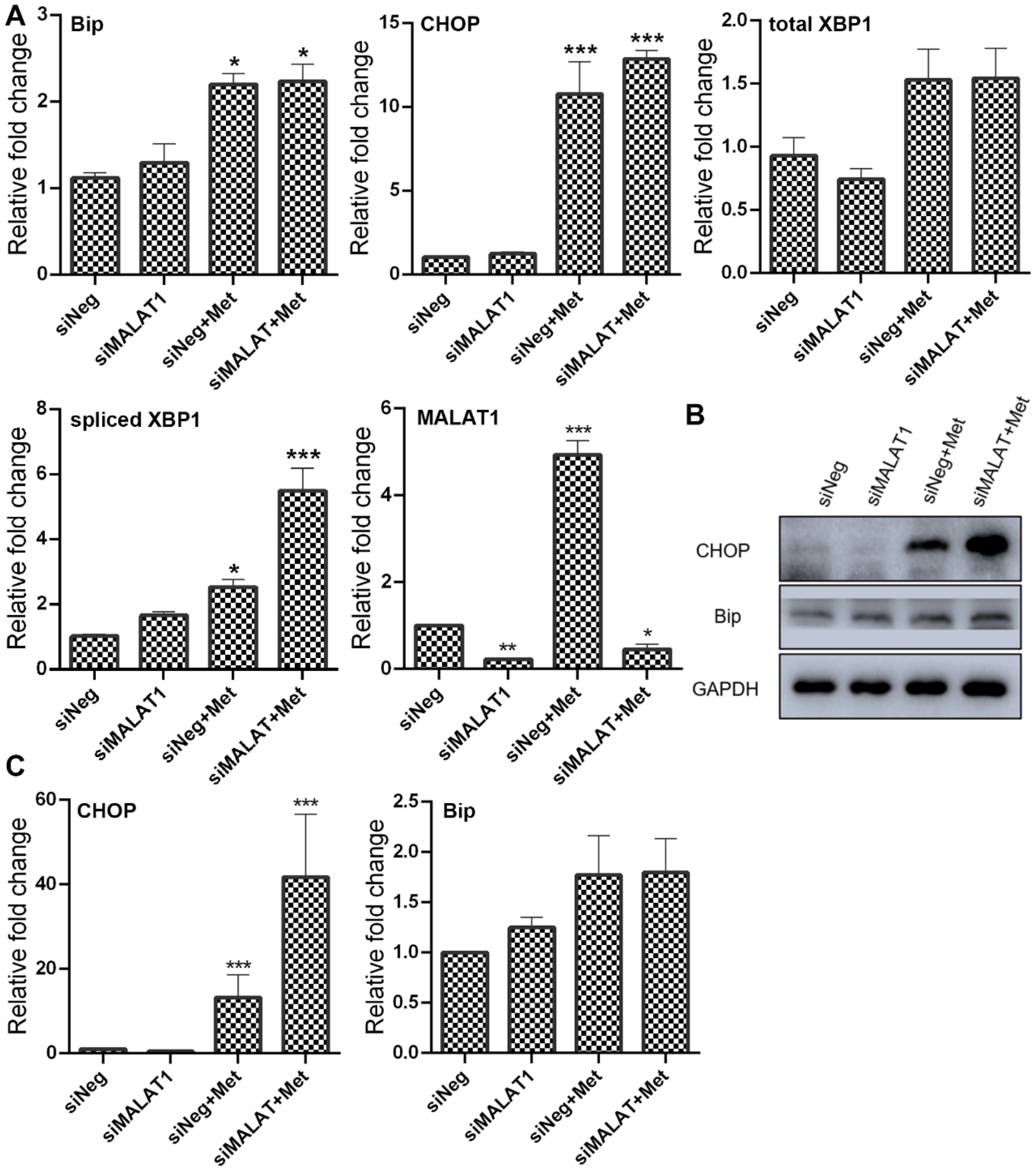

Figure S4. Densitometry in Western blot analysis was calculated by comparing with internal protein β-actin or GAPDH. *p<0.05 versus control, **p<0.01 versus control and ***p<0.001 versus control.

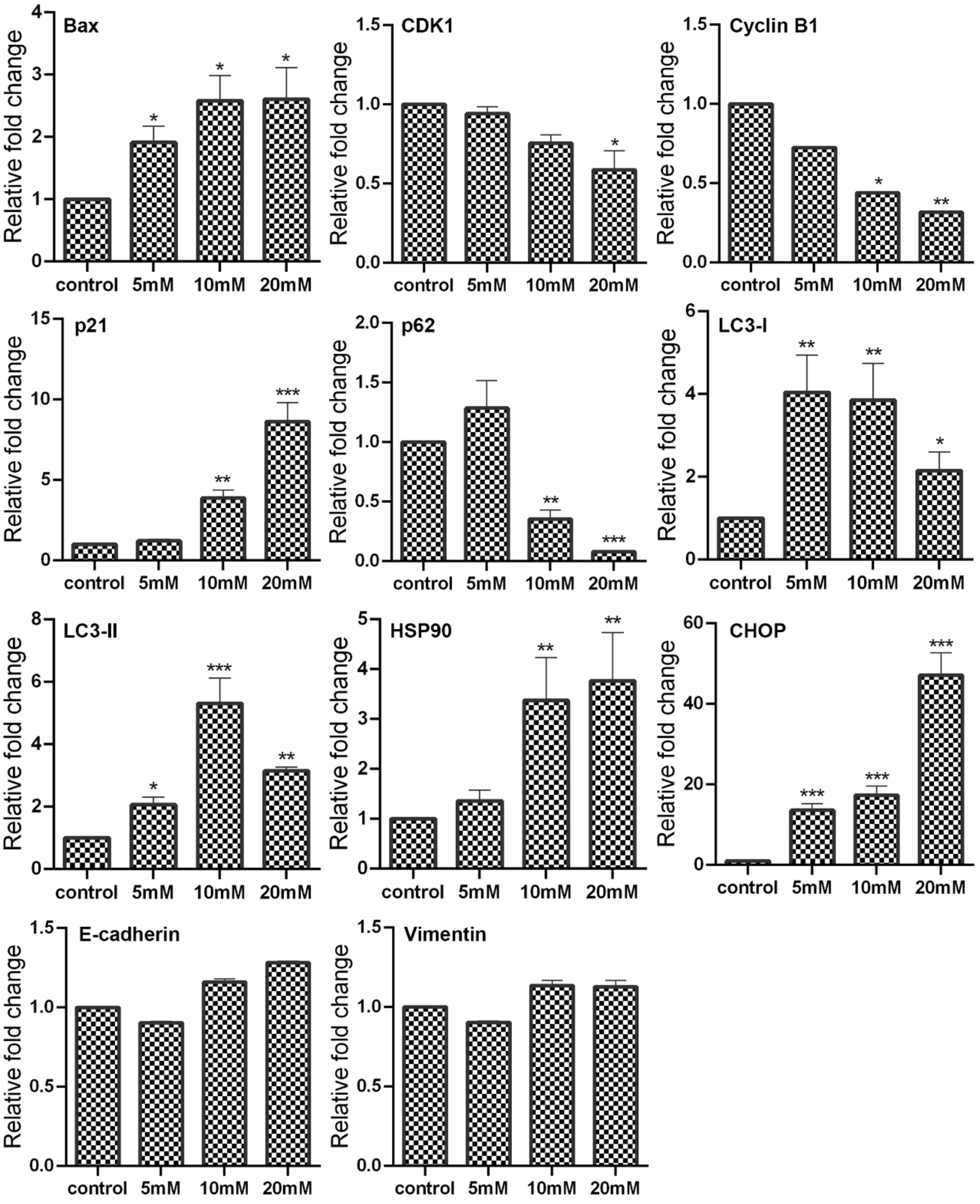

Figure S5. Densitometry in Western blot analysis was calculated by comparing with internal protein β-actin or GAPDH. *p<0.05 versus control, **p<0.01 versus control and ***p<0.001 versus control. siNeg, cells treated with negative control siRNA; siMALAT1, cells treated with MALAT1 siRNA; siNeg+Met, cells treated with negative control siRNA in combination with metformin; siMALAT+Met, cells treated with MALAT1 siRNA in combination with metformin.

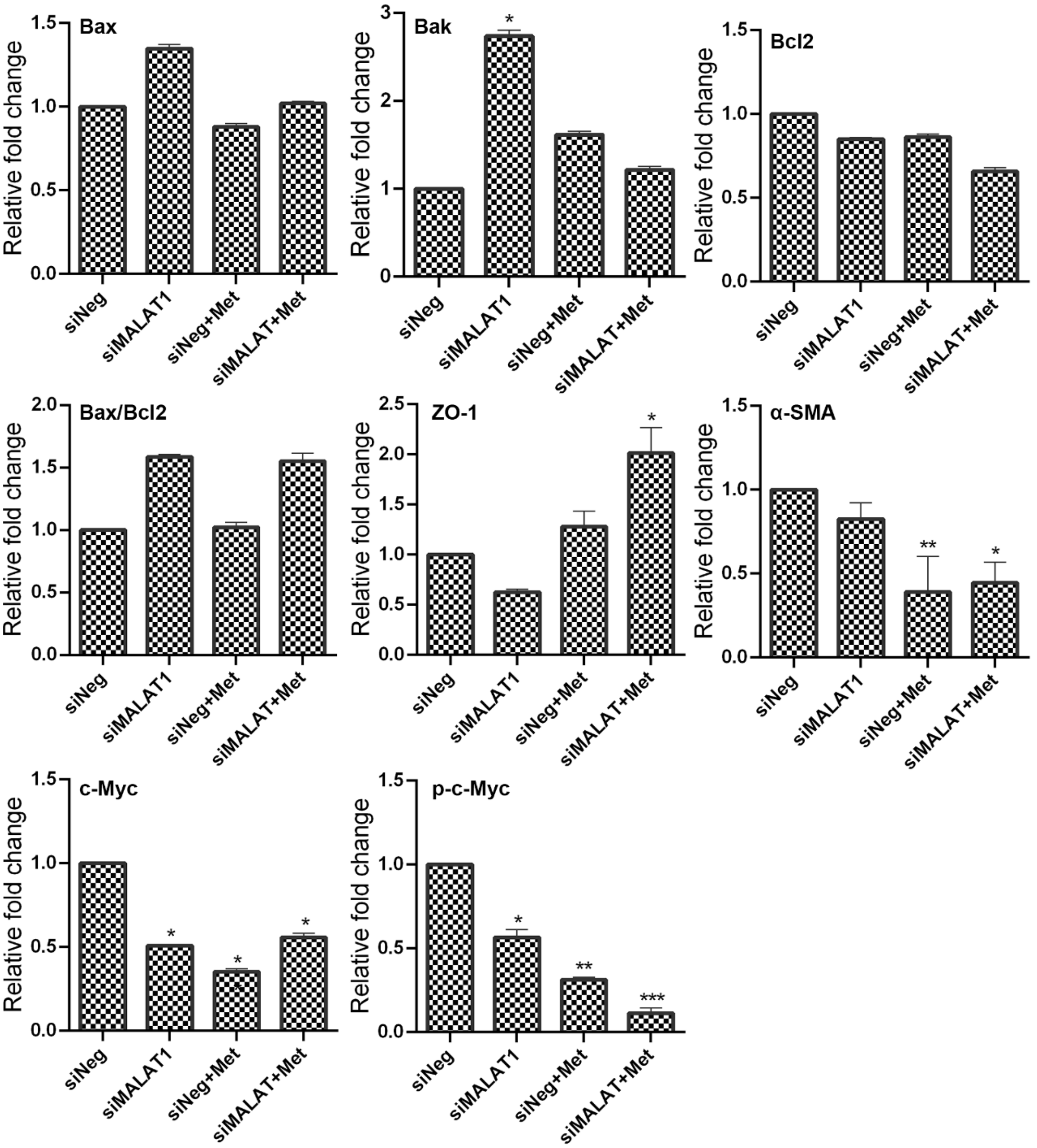

Figure S6. Densitometry in Western blot analysis was calculated by comparing with internal protein β-actin or GAPDH. *p<0.05 versus control, **p<0.01 versus control and ***p<0.001 versus control. siNeg, cells treated with negative control siRNA; siMALAT1, cells treated with MALAT1 siRNA; siNeg+Met, cells treated with negative control siRNA in combination with metformin; siMALAT+Met, cells treated with MALAT1 siRNA in combination with metformin.

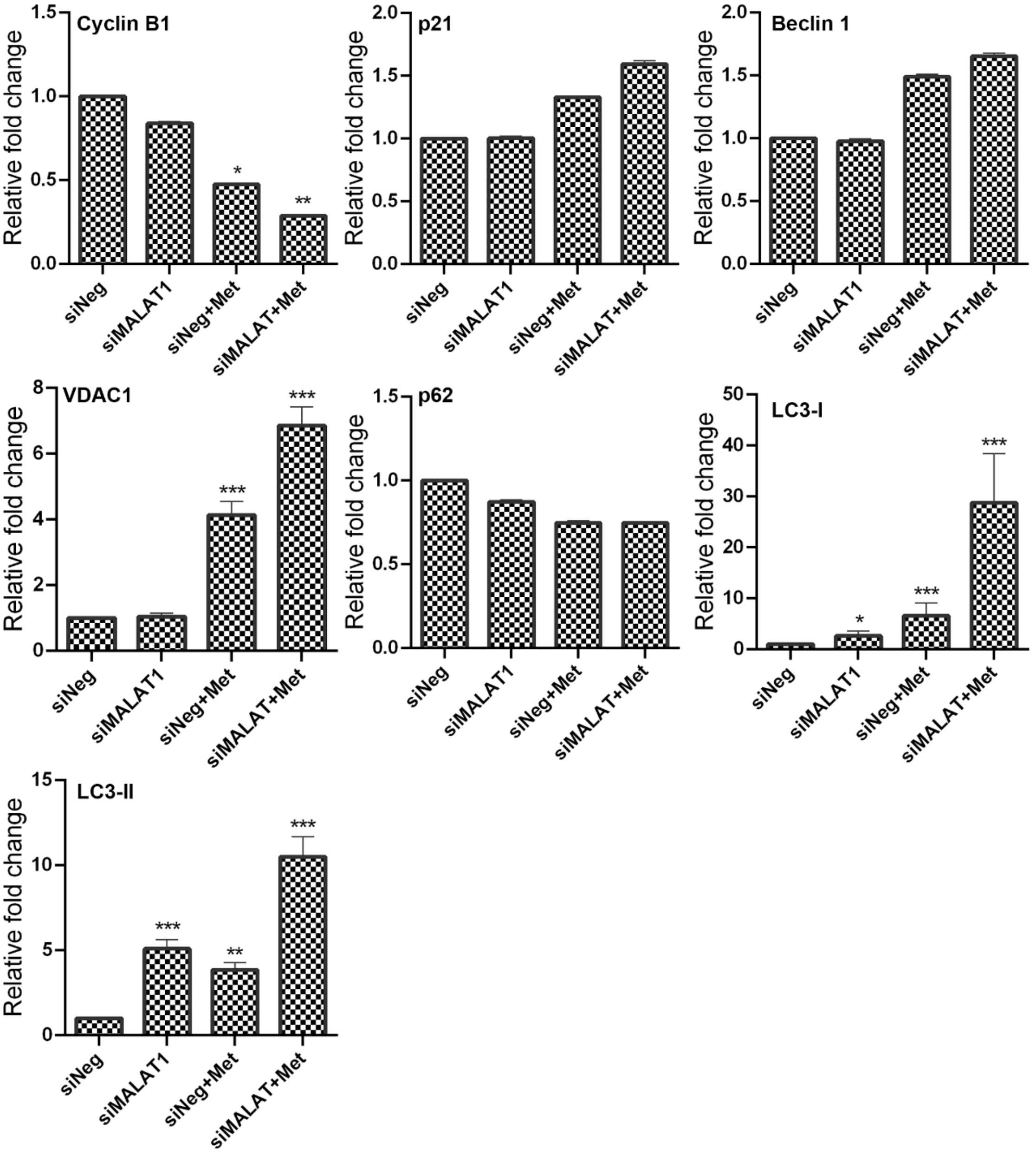

Supplement: Supplementary file 1 — Fig S1‐S6 [file JCMM-25-7135-s003.pdf]
