# Supplementary material for: lncRNA MALAT1 participates in metformin inhibiting the proliferation of breast cancer cell
Source: J Cell Mol Med. 2021 Jun 24;25(15):7135–45. doi: 10.1111/jcmm.16742 (PMC8335702; doi:10.1111/jcmm.16742)
Supplement: Supplementary file 2 — Tab S1 [file JCMM-25-7135-s002.docx]

**Supplementary Table S1. Primer sequences for qRT-PCR**

| **Genes** | **Forward** | **Reverse** |
| --- | --- | --- |
| CDK1 | GTCCGCAACAGGGAAGAACAG | CGAAAGCCAAGATAAGCAACTCC |
| p21 | GAGGCCGGGATGAGTTGGGAGGAG | CAGCCGGCGTTTGGAGTGGTAGAA |
| Cyclin B1 | CTGTTGGTTTCTGCTGGGTGTAG | CGCCTGCCATGTTGATCTTCG |
| Cyclin B2 | ACAAGTCCACTCCAAGTTTAGGC | CCAAGAGCAGAGCAGTAATCCC |
| Cyclin D1 | GCTGTGCATCTACACCGACAACTC | TTGCGGATGATCTGTTTGTTCTCCT |
| HRK | CCTACTGGCCTTGGCTGTG | TACAAGTTCCGCCTGCCG |
| TNFRSF10A | ACACCCAGCAAAGTGTGG | CCGACGACGACAAACTTG |
| TNFRSF10B | GACTATAGCACTCACTGGAATGACC | GTCATCGAAGCACTGTCTCAGAG |
| GRP78/Bip | ACGTGGAATGACCCGTCTGT | AACCACCTTGAACGGCAAGA |
| ATF4 | GTCAGTCCCTCCAACAACA | GGTGTCTTCCTCCTTTATGC |
| ATF6 | CTTTTAGCCCGGGACTCTTT | TCAGCAAAGAGAGCAGAATCC |
| CHOP | CAGAGCTGGAACCTGAGGAG | TGGATCAGTCTGGAAAAGCA |
| Total XBP1 | TTGTCACCCCTCCAGAACATC | TCCAGAATGCCCAACAGGAT |
| Spliced XBP1 | TGCTGAGTCCGCAGCAGGTG | GCTGGCAGGCTCTGGGGAAG |
| ATG3 | GCCGTTAAAGAGATCACACTGG | CATAGCCAAACAACCATAATCGTGG |
| ATG5 | CAGCTCTTCCTTGGAACATC | GGCTGTGGGATGATACTAATATG |
| BECN | GAAGACGTGGAAAAGAACCGC | CAGCCTGAAGTTATTGATTGTGC |
| LC3 | GATGTCCGACTTATTCGAGAGC | TTGAGCTGTAAGCGCCTTCTA |
| HSP90 | TTC AGA CAG AGC CAA GGT GC | CAA TGA CAT CAA CTG GGC AAT |
| MMP2 | GGAAAGCCAGGATCCATTTT | ATGCCGCCTTTAACTGGAG |
| MMP9 | TTGGTCCACCTGGTTCAACT | ACGACGTCTTCCAGTACCGA |
| E-cadherin | GGA TTG CAA ATT CCT GCC ATT C | AAC GTT GTC CCG GGT GTC A |
| Vimentin | GAC AAT GCG TCT CTG GCA CGT CTT | TCC TCC GCC TCC TGC AGG TTC TT |
| Fibronectin | TGA CCT TTT CTG GCT CGT CT | GTT CAG CAC AAA GGG CTC TC |
| Wnt3a | TGCATAGGCTCCTTCCTGTGG | TGGCTGGTGGGCTGAATTTC |
| Wnt5a | GAGTGCTCGCATCCTCAT | GCATGTCTTCAGGCTACA |
| β-catenin | GATTTGATGGAGTTGGACATGG | TGTTCTTGAGTGAAGGACTGAG |
| c-Myc | TCAGAGGTGCCACGTCTCC | TCTTGGCAGCAGGATAGTCCTT |
| MALAT1 | GACGAGTTGTGCTGCTATCTT | GATTCTGTGTTATGCCTGGTTAG |
| HOTAIR | CAGTGGGGAACTCTGACTCG | GTGCCTGGTGCTCTCTTACC |
| DICER1-AS1 | TGACCAGTCTTACCCCTCCT | CTGAAGCACCTGAAATGCG |
| LINC01121 | GGAAGAAGTGTGCTGTGCCA | CAACCCCTGACTCCTACACG |
| TUG1 | CTGAAGAAAGGCAACATC | GTAGGCTACTACAGGATTTG |
| PTTG3P | GGGGTCTGGACCTTCAATCAA | GCTTTAGGTAAGGATGTGGGA |
| H19 | AAGAAGGAGGTTTAGGGGATCG | CCGAGAAGATGTCACCTTTGCT |
| CDK1 | GTCCGCAACAGGGAAGAACAG | CGAAAGCCAAGATAAGCAACTCC |
| cyclinB2 | ACAAGTCCACTCCAAGTTTAGGC | CCAAGAGCAGAGCAGTAATCCC |
| cyclinB1 | CTGTTGGTTTCTGCTGGGTGTAG | CGCCTGCCATGTTGATCTTCG |
| CHOP | CAGAGCTGGAACCTGAGGAG | TGGATCAGTCTGGAAAAGCA |
| LC3 | GATGTCCGACTTATTCGAGAGC | TTGAGCTGTAAGCGCCTTCTA |
| RBBP4 | TTGATGCGTCACACTACGACAG | CAAGTCTGGGTTGCACTCTCC |
| RBBP7 | TTGAGTGGACATCTCCTAAGTG | CCTGGTGTCCCATATCATAAGT |
| G9a | TGACTGCGTGCTGTTATTCC | CGATTTCCCACCCCAAG |
| SET | GTCAAACGCAGAATAAAGCC | ATCATCCATATCGGGAACCAA |
| SETD1A | AAGGTGTACCGCTATGAT | CCAATATAGAACTCGTCCAG |
