# Supplementary material for: lncRNA MALAT1 participates in metformin inhibiting the proliferation of breast cancer cell
Source: J Cell Mol Med. 2021 Jun 24;25(15):7135–45. doi: 10.1111/jcmm.16742 (PMC8335702; doi:10.1111/jcmm.16742)
Supplement: Supplementary file 3 — Tab S2 [file JCMM-25-7135-s001.docx]

Table S2. List of primary antibodies applied in this study

| Antibody | brand | dilution | application |
| --- | --- | --- | --- |
| p21 | Bimake | 1:1000 | Western blotting |
| cyclinB1 | Bimake | 1:1000 | Western blotting |
| Bak | Bimake | 1:1000 | Western blotting |
| CDK1 | proteintech | 1:1000 | Western blotting |
| α-SMA | proteintech | 1:1000 | Western blotting |
| ZO-1 | proteintech | 1:1000 | Western blotting |
| CHOP | proteintech | 1:1000 | Western blotting |
| Bip | proteintech | 1:2000 | Western blotting |
| HSP90 | Bimake | 1:1000 | Western blotting |
| p62 | proteintech | 1:2000 | Western blotting |
| LC3 | proteintech | 1:1000 | Western blotting |
| Bax | Invitrogen | 1:1000 | Western blotting |
| Bcl2 | Bimake | 1:1000 | Western blotting |
| Vimentin | CST | 1:1000 | Western blotting |
| E-cadherin | CST | 1:1000 | Western blotting |
| BECN | proteintech | 1:2000 | Western blotting |
| VDAC | CST | 1:1000 | Western blotting |
| acH3K9 | CST | 1:1000 | Western blotting |
| acH3K18 | CST | 1:1000 | Western blotting |
| H3 | CST | 1:1000 | Western blotting |
| G9a | proteintech | 1:1000 | Western blotting |
| VPS4A | proteintech | 1:1000 | Western blotting |
| Wnt5a | CST | 1:1000 | Western blotting |
| β-catenin | CST | 1:1000 | Western blotting |
| β-actin | CST | 1:2000 | Western blotting |
| GAPDH | CST | 1:5000 | Western blotting |
